# Extended Prodromal Period in Herpes Zoster: A Case Report and Management Implications

**DOI:** 10.7759/cureus.79114

**Published:** 2025-02-16

**Authors:** Hidehiro Someko, Kazumori Takamoto, Yuki Kataoka

**Affiliations:** 1 Department of Healthcare Epidemiology, Graduate School of Medicine, Kyoto University, Kyoto, JPN; 2 Department of Systematic Reviewers, Scientific Research WorkS Peer Support Group (SRWS-PSG), Osaka, JPN; 3 Department of Internal Medicine, Kyoto Min-iren Asukai Hospital, Kyoto, JPN; 4 Department of Dermatology, National Hospital Organization Kyoto Medical Center, Kyoto, JPN; 5 Department of Dermatology, Kyoto Min-iren Asukai Hospital, Kyoto, JPN; 6 Department of Healthcare Epidemiology, Graduate School of Medicine/School of Public Health, Kyoto University, Kyoto, JPN; 7 Department of International and Community Oral Health, Graduate School of Dentistry, Tohoku University, Sendai, JPN

**Keywords:** dermatome, diagnostic and therapeutic challenge, neuropathic pain treatment, varicella-zoster virus, zoster sine herpete radicular pain

## Abstract

Herpes zoster typically presents with a prodromal phase of pain lasting three to five days before the appearance of characteristic vesicular lesions. We report a case of herpes zoster in an immunocompetent man in his 70s who experienced an unusually prolonged prodromal period of two weeks. The patient initially presented with radicular pain in the right lower extremity, leading to evaluation for lumbar spinal stenosis. After the appearance of vesicular lesions in the L4-S1 dermatomes, he was diagnosed with herpes zoster and treated with valacyclovir and pregabalin. Despite the delayed initiation of therapy, the patient's symptoms resolved completely without recurrence. This case demonstrates that prolonged prodromal periods can occur in immunocompetent individuals and highlights the importance of maintaining clinical suspicion for herpes zoster in cases of persistent radicular pain. Although our patient recovered well despite treatment delay, prompt initiation of antiviral therapy is recommended once herpes zoster is clinically suspected to ensure optimal outcomes.

## Introduction

Herpes zoster (HZ), commonly known as shingles, is a localized, painful vesicular eruption resulting from the reactivation of latent varicella-zoster virus (VZV) in sensory ganglia [1]. The disease typically affects older adults and immunocompromised individuals, with an estimated lifetime risk of 30% in the general population [2,3].

The pathophysiology of HZ explains its characteristic presentation and age distribution. Following primary infection (chickenpox), VZV establishes latency in sensory nerve ganglia. Reactivation occurs when cell-mediated immunity wanes, leading to viral replication and anterograde transport along sensory nerves to the skin. This process manifests clinically in the following two phases: an initial prodromal phase characterized by pain, burning, or tingling in the affected dermatome, followed by the emergence of characteristic vesicular lesions [4]. The duration of the prodromal phase may be influenced by factors such as the initial viral load in the ganglia, the rate of viral replication, and the host's immune response [5]. In some cases, vesicular lesions do not develop because viral transport to cutaneous tissue does not follow neuronal reactivation (zoster sine herpete) [6].

While the standard prodromal period typically lasts three to five days, atypical presentations with extended prodromes pose diagnostic challenges for clinicians. Cases where the interval between initial symptoms and cutaneous manifestations extends beyond two weeks are particularly noteworthy due to their potential for delayed diagnosis and treatment initiation. The literature documenting such prolonged prodromal periods is sparse, with only a handful of cases reporting prodromes lasting two to three weeks [7].

We present a case of herpes zoster with an unusually prolonged prodromal period of two weeks, highlighting the importance of maintaining clinical suspicion for HZ even when the presentation deviates from typical temporal patterns.

## Case presentation

**Figure 1:**
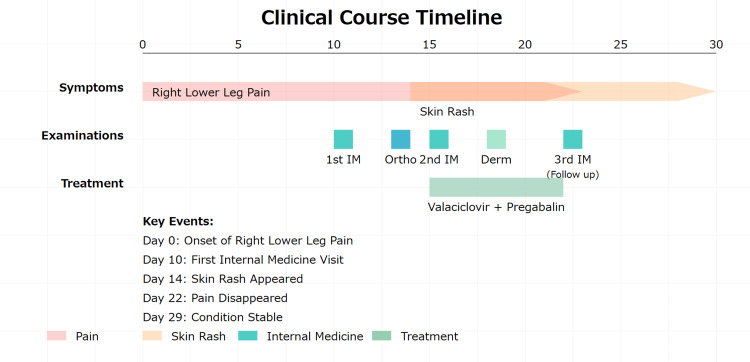
Clinical course timeline. IM: internal medicine; Ortho: orthpedics; Derm: dermatology

A 70-year-old man presented to the internal medicine department with a 10-day history of right lower extremity discomfort and intermittent tingling pain from the thigh downward. The complete clinical course is summarized in Figure 1.

The pain was described as dull and heavy, with occasional sharp sensations. Walking provided the most comfort, while forward bending was somewhat difficult. His medical history included bladder cancer treated with cystectomy eight years prior, intestinal obstruction requiring surgery two to three years ago, glaucoma, and cataract surgery. His regular medications included latanoprost eye drops and gastrointestinal medications (daikenchuto, mosapride citrate, and magnesium oxide). He had received three doses of COVID-19 vaccine and had no history of shingles vaccination.

Physical examination was unremarkable, and ankle-brachial index testing showed normal values (right: 1.17, left: 1.19), ruling out peripheral arterial disease. Suspecting an orthopedic condition, the patient was referred to the orthopedics department.

**Figure 2:**
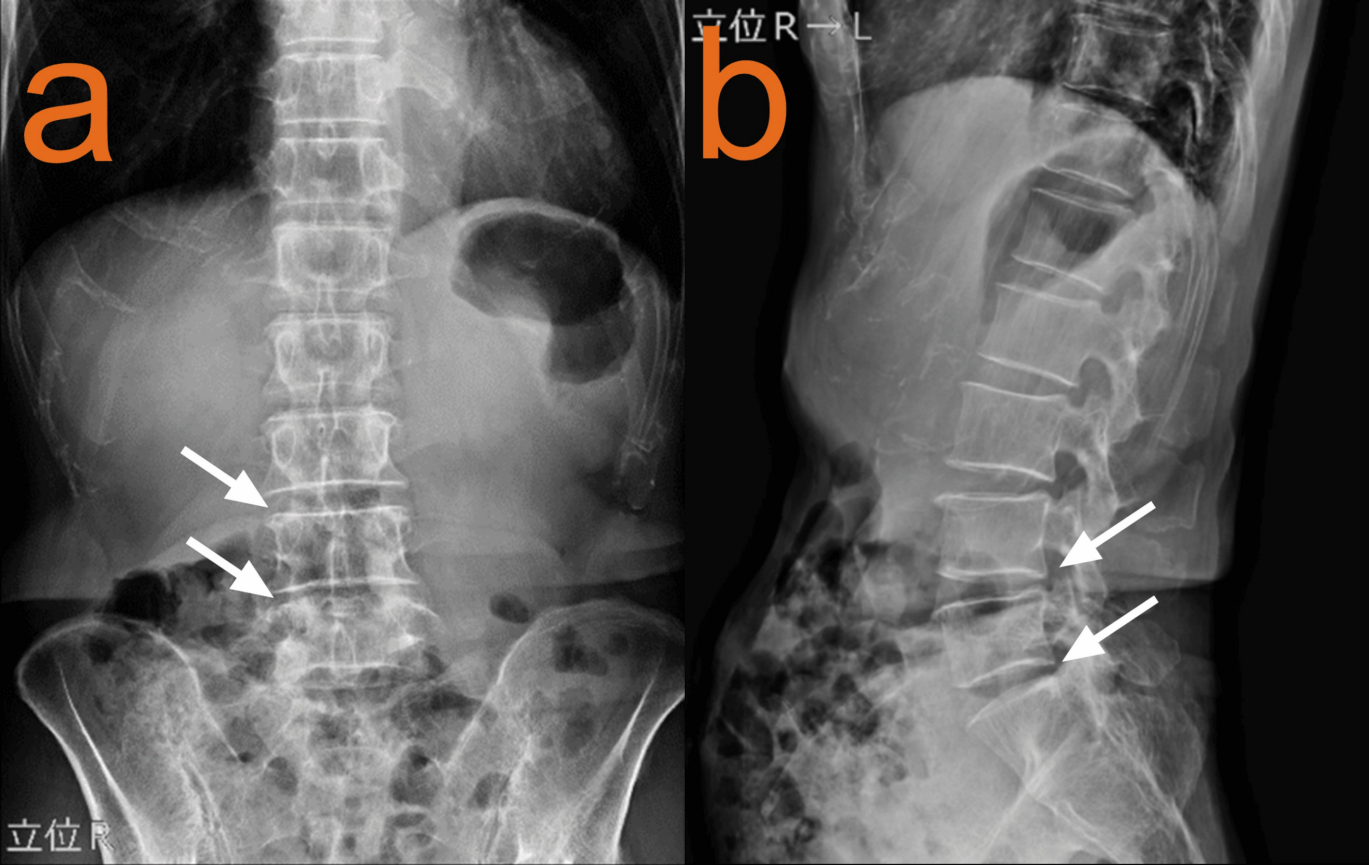
Lumbar spine x-ray (a: anteroposterior view, b: lateral view). Anteroposterior (a) and lateral (b) radiographs of the lumbar spine showing mild degenerative changes with disc space narrowing at L4/5 and L5/S1 levels. White arrows show a slight narrowing of the L4/5 and L5/S1 intervertebral disc spaces. While these findings would typically be considered age-appropriate incidental changes in asymptomatic patients, they initially led to the misattribution of the patient's symptoms to spinal pathology.

Three days later, at the orthopedics visit, physical examination revealed normal straight leg raising test bilaterally, with a full range of motion in the hip and knee joints. Plain radiographs showed slight narrowing of the L4/5 and L5/S1 intervertebral spaces (Figures 2a, 2b).

**Figure 3:**
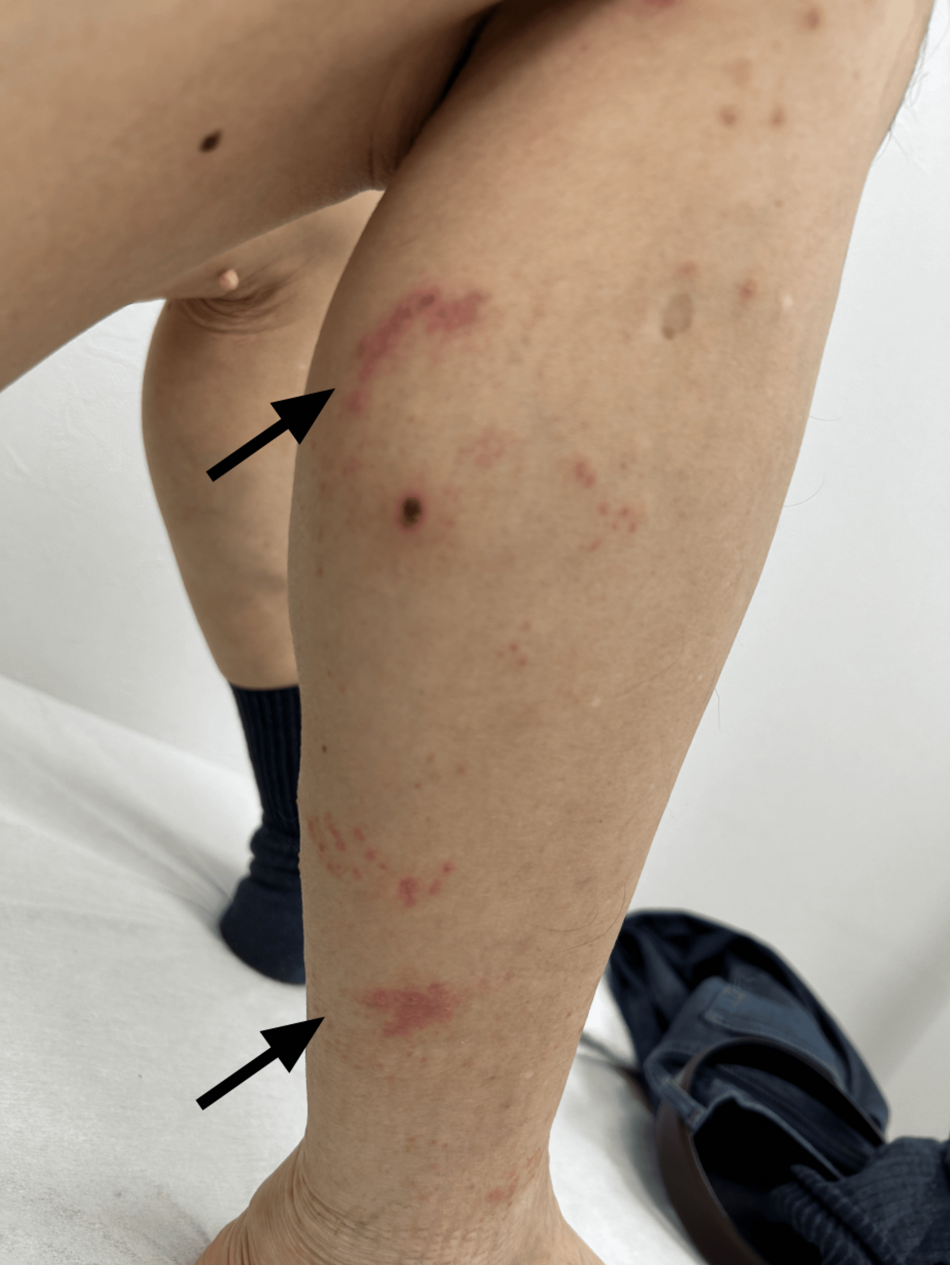
Grouped vesicular eruptions on erythematous base (black arrows) distributed along the right lower leg in a dermatomal pattern (L4–S1). The eruption occurred after 14 days of pain, demonstrating the prolonged prodromal period discussed in this case. Note the clustered vesicles confined to specific dermatomes, a pattern that helped confirm the diagnosis after the initial misattribution of symptoms to lumbar radiculopathy. Although the morphology of individual lesions might suggest contact dermatitis or arthropod bites, these diagnoses were excluded based on the characteristic dermatomal distribution and the absence of any relevant exposures or incidents.

An MRI was scheduled for further evaluation. The following day, the patient noticed skin lesions on his right lower extremity. On day five, he returned to the internal medicine department due to severe pain that had intensified overnight, disturbing his sleep. Physical examination revealed vesicles with erythema on the lateral aspect of the right lower leg, extending to the dorsum and heel, corresponding to the L4-S1 dermatomes (Figure 3).

His vital signs showed a blood pressure of 184/107 mmHg, heart rate of 89 beats per minute, and body temperature of 36.6°C. Laboratory tests showed normal complete blood count and inflammatory markers, with a white blood cell count of 4,400/μL and C-reactive protein of 0.11 mg/dL (Table 1).

**Table 1:** The results of laboratory tests on day five. HbA1c: hemoglobin A1c; NGSP: National Glycohemoglobin Standardization Program; CRP: C-reactive protein; BUN: blood urea nitrogen; AST: aspartate aminotransferase; ALT: alanine aminotransferase; LDH: lactate dehydrogenase; CK: creatine kinase; ALP: alkaline phosphatase; γ-GTP: gamma-glutamyl transpeptidase

Laboratory test	Patient value	Reference range
Blood glucose	103 mg/dL	70-110 mg/dL
HbA1c (NGSP)	5.10%	4.6-6.2%
CRP	0.11 mg/dL	<0.30 mg/dL
Total protein	7.5 g/dL	6.5-8.2 g/dL
Albumin	4.1 g/dL	3.5-5.2 g/dL
BUN	12.3 mg/dL	8-20 mg/dL
Creatinine	0.68 mg/dL	0.6-1.1 mg/dL
Uric acid	2.6 L mg/dL	3.0-7.0 mg/dL
AST	20 U/L	13-33 U/L
ALT	15 U/L	8-42 U/L
LDH	162 U/L	120-246 U/L
CK	83 U/L	45-163 U/L
ALP	67 U/L	38-113 U/L
γ-GTP	17 U/L	10-47 U/L
Total bilirubin	1.0 mg/dL	0.3-1.2 mg/dL
WBC	4,400/μL	3,500-9,000/μL
Hemoglobin	13.6 g/dL	13.0-17.0 g/dL
Hematocrit	40.10%	40.0-50.0%
Platelet	19.3 ×10⁴/μL	15.0-35.0 ×10⁴/μL

**Figure 4:**
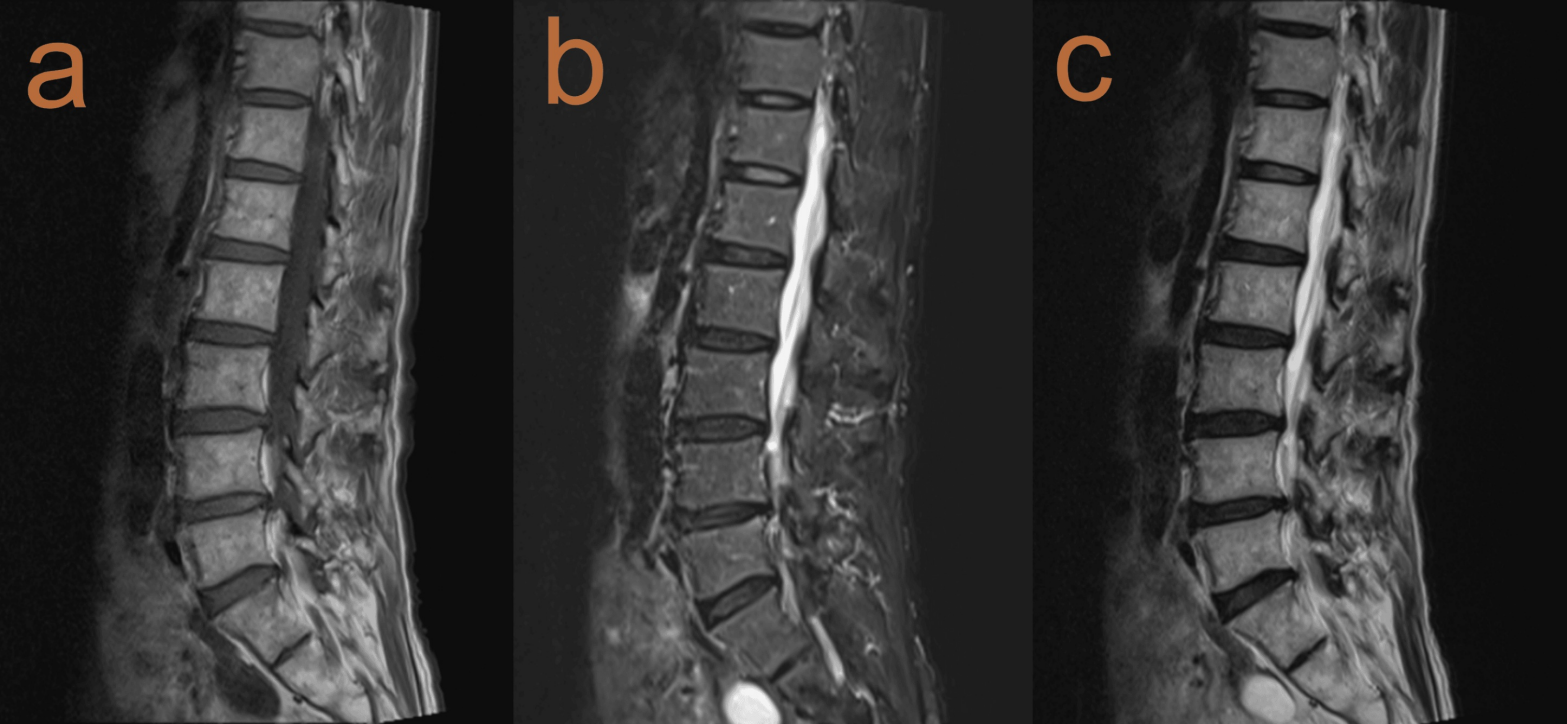
Lumbar spine magnetic resonance imaging showing no significant pathology to explain the patient's radicular symptoms. (a) T1-weighted sagittal view, (b) T2-weighted sagittal view, and (c) fat-suppressed T2-weighted sagittal view demonstrate normal vertebral alignment and disc heights without evidence of nerve root compression or significant disc herniation.

Despite the unusually long prodromal period, herpes zoster was diagnosed based on the clinical presentation, and the patient was started on oral valacyclovir and pregabalin. Three days later, a dermatology consultation confirmed the diagnosis of herpes zoster. The scheduled MRI was performed on day 10, showing no significant abnormalities (Figures 4a-4c).

At the follow-up visit to internal medicine on day 12, the pain had completely resolved, and both valacyclovir and pregabalin were discontinued. The patient remained stable at day 19, with no relapse of pain, supporting the final diagnosis of herpes zoster.

## Discussion

This case highlights the following two important clinical aspects of herpes zoster: the possibility of a prolonged prodromal period and the effectiveness of standard therapy even when initiated after a delayed diagnosis. When evaluating patients with persistent radicular or neuropathic pain, clinicians should maintain a high index of suspicion for herpes zoster, even in the absence of skin manifestations for periods longer than the typical three- to five-day prodrome. While prolonged prodromal periods have been primarily reported in immunocompromised patients, the present case demonstrates that this atypical presentation can occur in immunocompetent individuals as well [6]. Our patient was initially evaluated for lumbar spinal stenosis due to the prolonged radicular symptoms, leading to unnecessary imaging studies. In fact, these two entities are difficult to distinguish because they affect anatomically adjacent areas - shingles involving the sensory ganglion and compression radiculopathy affecting its proximal portion - and this close proximity results in overlapping signs and symptoms [8]. Although large epidemiologic studies are lacking for clear discrimination of these entities, some case reports suggest that a negative straight leg raise (SLR) test and the presence of allodynia are more indicative of shingles than compression radiculopathy [8-10]. In addition, multiple segmental involvement may be a valuable clinical clue suggesting shingles, as this pattern is relatively uncommon in typical radiculopathy cases as seen in the present case.

The favorable outcome in our patient, despite delayed treatment initiation, should not be interpreted as support for watchful waiting in suspected herpes zoster cases. Although our patient recovered well with standard therapy despite the unusually prolonged 14-day prodromal period, several important considerations warrant prompt treatment initiation once herpes zoster is clinically suspected. First, early antiviral therapy is well-established to reduce the risk of post-herpetic neuralgia and accelerate rash healing [11]. Second, while the existence of zoster sine herpete (herpes zoster without rash) highlights that skin manifestations are not always present, diagnosis of herpes zoster should be considered when patients present with progressive dermatomal pain, particularly when imaging studies fail to reveal alternative explanations such as lumbar radiculopathy [12]. This is especially relevant in cases involving the lower extremities, where herpes zoster can mimic compression radiculopathy [13]. While PCR testing remains the gold standard for diagnosis, its limited availability and cost considerations make clinical diagnosis based on characteristic features a practical necessity in many settings. Direct immunofluorescence testing can detect VZV antigens from vesicular lesions, but this diagnostic method is only applicable after the appearance of skin manifestations and therefore cannot aid in early diagnosis during the prodromal period [14]. VZV IgG antibodies primarily indicate past infection or vaccination rather than active disease, while VZV IgM testing lacks reliability in adults with herpes zoster reactivation, as it is more commonly elevated in primary varicella infection (chicken pox) [15]. Therefore, we recommend considering initiating antiviral therapy when herpes zoster is strongly suspected based on rapidly progressive dermatomal pain without clear alternative etiology, even in cases with prolonged prodromal periods.

The unusually prolonged prodromal period in the present case warrants pathophysiological consideration. While the typical prodromal phase of herpes zoster lasts three to five days, several factors may contribute to extended periods. Generally, the duration of the prodromal phase reflects the complex interplay between viral reactivation dynamics and host immune response. The initial viral load in the ganglia, rate of viral replication, and efficiency of the host's immune response all influence this timeline [5]. In the present case involving the lower extremity, the extended prodromal period might be attributed to the anatomical distance between the affected sensory ganglion and the eventual site of cutaneous manifestation.

## Conclusions

This case demonstrates that herpes zoster can present with an unusually prolonged prodromal period even in immunocompetent patients. Although our patient achieved symptom resolution with standard antiviral therapy despite delayed treatment, clinicians should not wait for the appearance of skin lesions before initiating therapy when herpes zoster is clinically suspected, in order to minimize the risk of postherpetic neuralgia. The possibility of zoster sine herpete and the well-established benefits of early antiviral therapy underscore the importance of prompt treatment initiation based on clinical suspicion, even in cases with atypical presentations or prolonged prodromal periods. To differentiate between shingles and orthopedic conditions, clinical signs such as allodynia, reverse SLR test findings, and multiple segmental involvement can be helpful diagnostic indicators. In these cases, prompt treatment may benefit the patient.

## References

[1] Gershon AA, Breuer J, Cohen JI (2015). Varicella zoster virus infection. Nat Rev Dis Primers.

[2] Brisson M, Edmunds WJ, Law B (2001). Epidemiology of varicella zoster virus infection in Canada and the United Kingdom. Epidemiol Infect.

[3] Forbes HJ, Bhaskaran K, Thomas SL, Smeeth L, Clayton T, Langan SM (2014). Quantification of risk factors for herpes zoster: population based case-control study. BMJ.

[4] Straus SE, Ostrove JM, Inchauspé G, Felser JM, Freifeld A, Croen KD, Sawyer MH (1988). NIH conference. Varicella-zoster virus infections. Biology, natural history, treatment, and prevention. Ann Intern Med.

[5] Devin A, Renoult E, Roger M, Hébert MJ (2018). Herpes zoster in kidney transplant recipients: detection of VZV DNA in blood during the prodromal phase. Transplant Direct.

[6] Sahra S, Jahangir A, Glaser A, Mobarakai N, Jahangir A (2021). Case report: aseptic meningitis secondary to varicella-zoster virus (VZV) without an exanthem post MMR vaccination. BMC Infect Dis.

[7] Zerngast WW, Paauw DS, O'Connor KM (2013). Varicella zoster with extended prodrome: a case series. Am J Med.

[8] Kim KD, Lee DJ (2015). Commentary on: "postoperative shingles mimicking recurrent radiculopathy after anterior cervical diskectomy and fusion". Global Spine J.

[9] Mallepally AR, Mahajan R, Rustagi T, Marathe NA, Chhabra HS (2020). Varicella-zoster radiculitis mimicking sciatica: a diagnostic dilemma. Asian J Neurosurg.

[10] Rhyu KW, Shin JH, Kim YC, Cho SH, Kwon GH, Lee HY (2021). Prevesicular herpes zoster lumbar radiculopathy with transient motor paresis: a case report. Medicine (Baltimore).

[11] Lang P, Hasso Y, Michel JP (2009). Stop shingles in its tracks. J Fam Pract.

[12] Zhou J, Li J, Ma L, Cao S (2020). Zoster sine herpete: a review. Korean J Pain.

[13] Burkman KA, Gaines Jr RW, Kashani SR, Smith RD (1988). Herpes zoster: a consideration in the differential diagnosis of radiculopathy. Arch Phys Med Rehabil.

[14] Coffin SE, Hodinka RL (1995). Utility of direct immunofluorescence and virus culture for detection of varicella-zoster virus in skin lesions. J Clin Microbiol.

[15] Koskiniemi M, Piiparinen H, Rantalaiho T (2002). Acute central nervous system complications in varicella zoster virus infections. J Clin Virol.

